# Organoid, organ-on-a-chip and traditional Chinese medicine

**DOI:** 10.1186/s13020-025-01071-8

**Published:** 2025-02-12

**Authors:** Jiayue Yang, Yu Jiang, Mingxing Li, Ke Wu, Shulin Wei, Yueshui Zhao, Jing Shen, Fukuan Du, Yu Chen, Shuai Deng, Zhangang Xiao, Wen Yuan, Xu Wu

**Affiliations:** 1 Cell Therapy & Cell Drugs of Luzhou Key Laboratory, Department of Pharmacology, School of Pharmacy, Southwest Medical University, Luzhou, 646000 China; 2 Department of Gerontology, The Affiliated Traditional Chinese Medicine Hospital, Southwest Medical University, Luzhou, 646000 China; 3 South Sichuan Institute of Translational Medicine, Luzhou, 646000 China; 4 Gulin County Hospital of Traditional Chinese Medicine, Luzhou, 646500 China; 5 School of Pharmacy, Sichuan College of Traditional Chinese Medicine, Mianyang, 621000 Sichuan China; 6 Departments of Paediatrics & Paediatric Care, Luzhou People’s Hospital, Luzhou, 646000 Sichuan China

**Keywords:** Traditional Chinese medicine, Organoids, Organ-on-a-chip, Co-culture

## Abstract

In the past few years, the emergence of organoids and organ-on-a-chip (OOAC) technologies, which are complementary to animal models and two-dimensional cell culture methods and can better simulate the internal environment of the human body, provides a new platform for traditional Chinese medicine (TCM) studies. Organoids and OOAC techniques have been increasingly applied in the fields of drug screening, drug assessment and development, personalized therapies, and developmental biology, and there have been some application cases in the TCM studies. In this review, we summarized the current status of using organoid and OOAC technologies in TCM research and provide key insights for future study. It is believed that organoid and OOAC technologies will play more and more important roles in research and make greater contributions to the innovative development of TCM.

## Introduction

Traditional Chinese medicine (TCM) has evolved for a long history and has a unique cultural background. At present, research on TCM is faced with complex and diverse problems. Compared with chemical drugs and biological products, the scientific and technological requirements involved in the research process of TCM are very high, and the technical difficulties of research are even greater. Modern TCM research faces the following problems. First, natural medicines come from a wide range of sources and contain complex and diverse chemical components [1]. There are a large number of trace components in them. Although their content is low, they may have important biological activities. The key features of TCM,  including multiple active components,  result in complex network interactions with multi-targets in different systems in human. Second, the screening of natural active ingredients is also facing difficulties. There is a lack of efficient models for the screening of active ingredients. Existing screening models cannot fully simulate the complex physiological and pathological states of the human body. The characteristics of the multi-target action of TCM mentioned above are also a major difficulty in screening active natural ingredients. Third, the current safety evaluation of TCM also faces many challenges. The use of TCM is often long-term, and short-term safety evaluation may not be able to find the potential chronic toxicity and carcinogenesis of drug ingredients. And the responsiveness of TCM in different subject groups may vary. Last but not least, TCM often appears in the form of  compound prescription or is used simultaneously with other chemical drugs. At present, the research on the compatibility mechanism of TCM herbs and the interaction between various TCM and chemical drugs is not deep enough. Modern pharmacology has focused on the TCM study with the help of two-dimensional (2D) monolayer cell models and animal models [2–6] to evaluate its efficacy and mechanism. However, there are still key limitations in the application of these techniques in TCM research, including the lack of cell heterogeneity and cell-to-cell interaction, species differences, and lack of clinical-relevant characteristics. To better promote the research and development of TCM, it is necessary to continuously propose innovative research methods to enhance the analysis and identification of active components and accurately evaluate the efficacy, safety and mechanisms of TCM. In the past few years, the emergence of organoid and organ-on-a-chip (OOAC) technologies, which are complementary to animal models and 2D cell culture methods and can better simulate the internal environment of the human body [7], provides a new platform for TCM studies.

Organoids are cells grown in a three-dimensional (3D) environment that are capable of forming tissues and differentiating into functional cell types with the physiological structure and function of organs in the body. Therefore, organoids are also known as microorganisms [8]. Organoids can be derived from induced pluripotent stem cells (iPSCs), embryonic stem cells (ESCs), and neoplastic or adult stem cells (AdSCs), which are capable of self-renewal and sustained differentiation and exhibit histological structures and functional roles similar to those of organs in vivo [9]. Currently, there are several in vitro organoid models for different human internal organs such as the brain, lung, thyroid, stomach, small intestine, liver, kidney, etc. By simulating the internal environment of the human body, organoid systems can more realistically reflect the condition of organs in different environments, which helps to improve the reliability and validity of the research.

Huh and others introduced the concept of “organ chips” in a 2010 article “Lung-on-a-chip” [10]. OOAC is a microfluidic-based cell culture device capable of mimicking the physiology and function of human tissues and organs on a small-scale chip [11]. As a microfluidic chip, OOAC is usually fabricated using silicon-based organic polymer dimethylsiloxane (PDMS) using soft lithography technology [12]. The core of microfluidic technology makes it possible to manipulate and utilize microfluidics through microtubules and valves embedded in the chip. OOAC integrates living human cells with the cellular microenvironment and can mimic physiological homeostasis at the organ function level under pathological conditions [13]. Organ chips enable the investigation of interactions between cells and tissues, and can simulate fluid flow in the body, which is crucial for all cells and tissues in the body. For example, lung organ chips can reconstruct the microstructure of alveolar capillary units on microfluidic chips, simulate the alveolar-air–water interface, accurately manipulate the lung microenvironment, and analyze the physiological and pathological effects of factors such as tension, shear force, and pressure on the lungs [14]. This accurate microenvironment simulation is not possible with other in vitro models.

Organoids and OOAC techniques have been increasingly applied in the fields of drug screening, drug assessment and development, personalized therapies, and developmental biology [15]. Notably, the emergence of organoid and OOAC technologies, displaying some advantages over existing techniques, offers a new perspective for TCM studies, and there have been many application cases in this area. Therefore, in this review, we aim to summarize the current status of using organoid and OOAC technologies in TCM research and provide insights for future study.

## An overview of the application of organoids and OOAC

**Fig. 1:**
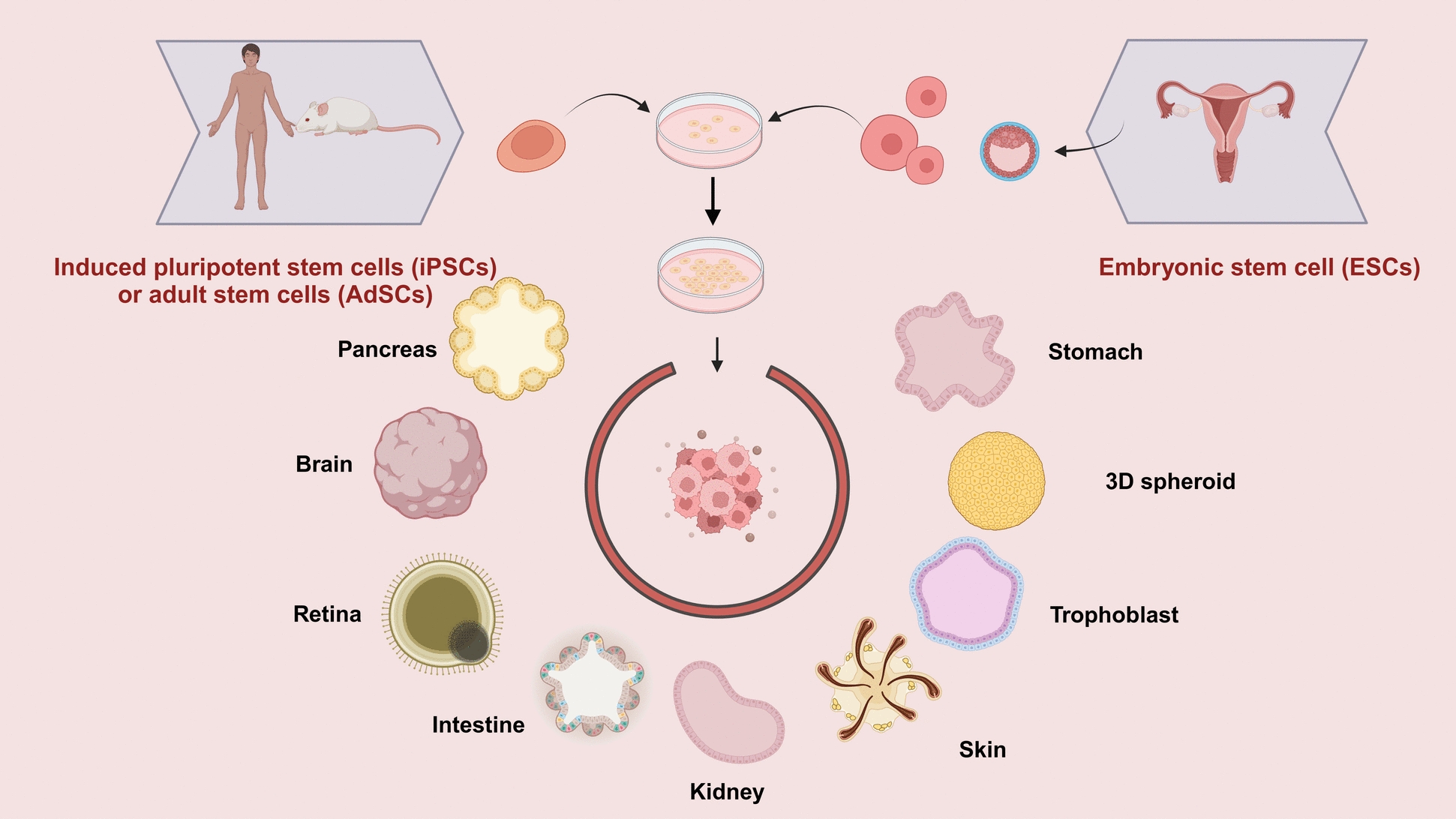
Organoids can be induced by human or animal embryonic stem cells (ESCs), pluripotent stem cells (iPSCs), or adult stem cells (AdSCs). The specific generation of target organoid models requires the addition of corresponding inducing factors

In the last few years, significant advancements have been achieved in the field of organoid research. Currently, a wide array of organoid models have been developed for various purposes, including those from varied tissues of brain [11], retina [16], cerebellum [17], hippocampus [18], lung [19], thyroid [20], stomach [21], liver [22], kidney [23], etc*.* (Fig. 1). Either iPSCs, ESCs, or AdSCs can be used (Fig. 1). Stem cells can maintain self-renewal and development throughout the adult body, and can proliferate and differentiate into a variety of cells, tissues, or organs, which is essential for maintaining tissue homeostasis and tissue repair [24]. In 2009, Sato et al*.* first used adult intestinal stem cells to form 3D intestinal organoids in matrix gel, and self-organized differentiation formed intestinal crypt villi structure [8]. AdSCs-derived organoids are directly produced from postnatal or adult tissues, which are inoculated into a medium containing selected growth factors required for culture. After the cells proliferate, differentiate, and migrate, spontaneous tissues form organoids with similar structure and complexity to internal organs. AdSCs-derived organoids can also be constructed from patient-specific tissues for disease modeling and precision medicine [25]. The development of ESC/iPSC-derived organoids necessitates a variety of growth factors or inhibitors to induce directional differentiation, and the obtained organoids are suitable for obtaining some difficult-to-obtain internal organ tissues and for simulating the growth and development process of internal organs [25]. Organoids can also be used in regenerative medicine. For example, bone marrow-derived mesenchymal stem cells (BM-MSCs) can be used in stem cell therapy and are also widely used in regenerative medicine. However, there are still some difficulties in the current methods for obtaining BM-MSCs, such as insufficient primary cell yield, long in vitro expansion time, and poor differentiation ability after passage. The number of mesenchymal stem cells harvested from bone organoids is high, the purity is high, the dryness is strong, and the time required is short, which provides a better solution for stem cell research and tissue engineering [26].

**Fig. 2:**
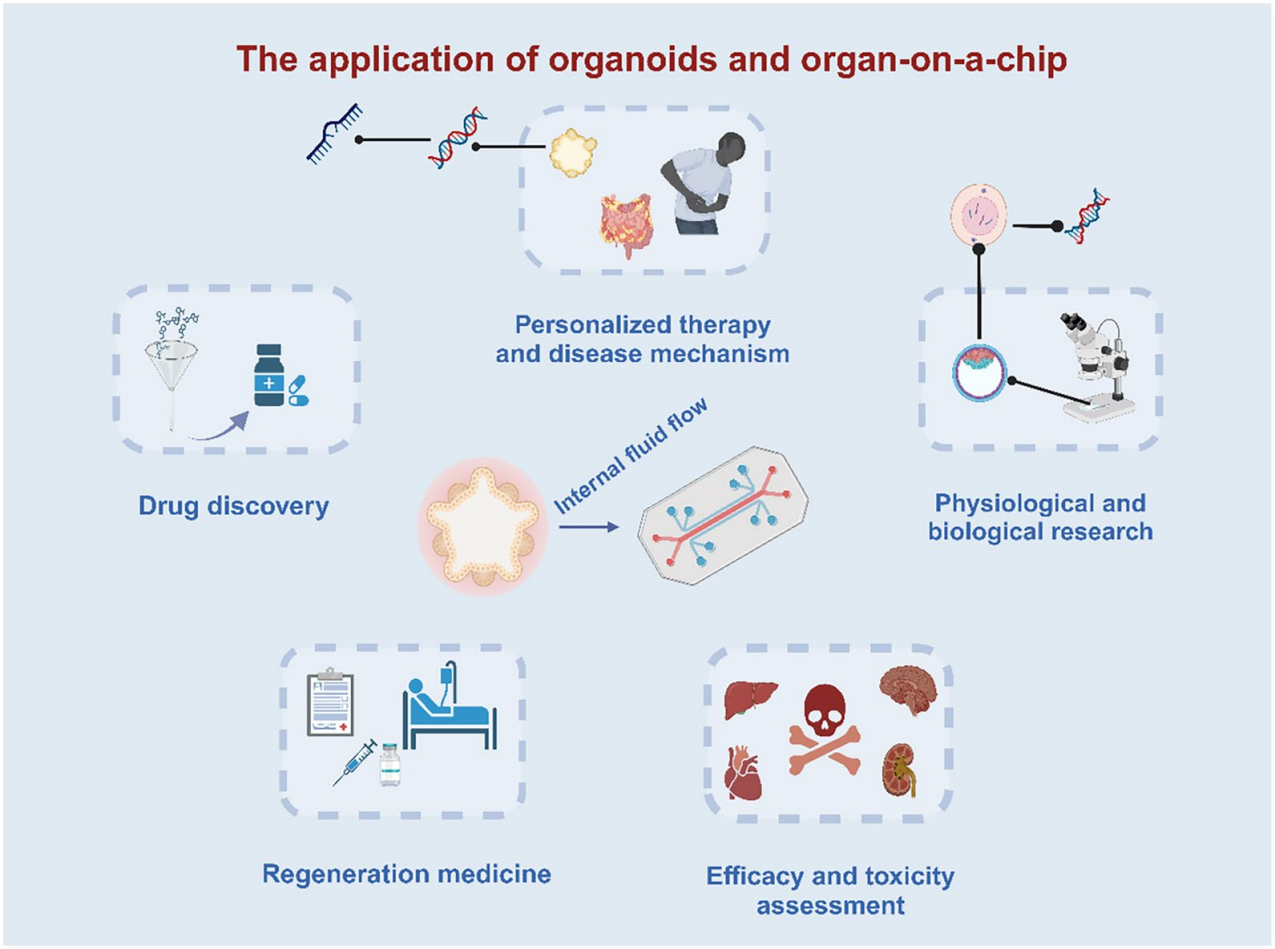
Organoids and organ-on-a-chip (OOAC) can be used for drug discovery, efficacy and toxicity assessment, personalized therapy, disease mechanism, and basic physiology research. Organoids can also be used in regenerative medicine. Compared to organoids, the OOAC technique can simulate fluid flow in the body

Organoids, which exhibit a greater cellular diversity and genomic stability compared to traditional 2D cell cultures, accurately replicate the intricate cellular interactions within the extracellular environment. Organoid technology is mainly used in the fields of infectious diseases, hereditary diseases, new drug discovery and toxicological evaluation, personalized therapies, regenerative medicine, and gene therapy [27] (Fig. 2). Among them, organoid models play an important role in the fields of new drug development and toxicity testing. By replicating the physiological conditions of the human body, including temperature, humidity, oxygen levels, and other environmental variables, organoid systems can better emulate the state of organs in various settings, thereby enhancing the credibility and relevance of scientific investigations. Leveraging organoids for drug testing and assessment enhances the effectiveness and precision of research and development endeavors. Meanwhile, in terms of drug toxicity testing, organoids can simulate the response and metabolism of human organs to drugs to more accurately assess the degree of harm caused by drugs and chemicals to the human body [28]. The use of organoid screening of drug components can also reduce the number of experimental animals. Traditional drug testing and the construction of disease models often require a large number of animals for experiments, which not only consumes a large number of resources but also involves animal welfare and ethical issues. The emergence of organoids can reduce the use of animals to a certain extent to achieve rapid screening and evaluation of new drug research.

OOAC is based on a microfluidic chip. Microfluidics provide flexibility in terms of liquid composition, flow rate, temperature, etc., thus facilitating the different mechanical properties required in various experiments [1]. Organ chips require dynamic mechanical stress to simulate the daily stress of human organs, such as blood pressure and pulmonary pressure. Microfluidic technology uses elastic porous membranes to simulate this periodic mechanical stress. The fluid shear force realizes the dynamic culture of cells through micropump perfusion, and the concentration layer acts as a laminar flow to generate stable biochemical signals, including angiogenesis, invasion, migration, etc. By manipulating the microvalves and micropumps to change the flow rate and channel shape, it is able to simulate the complex physiological and pathological processes in the human body [29]. OOAC simulates in vivo cellular tissues and organs that are not possible with traditional 2D and 3D cell cultures. This model enables real-time in vitro analysis of the biochemical, genetic, and metabolic activities of living cells in a functional tissue and organ environment [30].

OOAC is widely used in the field of drug screening to evaluate the safety and efficacy of new drugs more accurately and efficiently by simulating physiological and pathological conditions, as well as interactions between cells and tissues [10]. The emergence of this technology greatly accelerates the process of drug development and provides a more reliable basis for clinical drug therapy (Fig. 2). First, organ chips consist of artificially fabricated microfluidic systems and human cells, which can simulate the interactions between cells as well as between cells and tissues and thus accurately simulate the human physiological environment [31]. Through this simulation, researchers can more accurately understand the effects of new drugs on the body and assess the pharmacokinetics/metabolism, toxicity, and efficacy of drugs. Second, the results based on OOAC can be more clinically relevant if organoids or OOAC are derived from patients or healthy subjects. Traditional drug development requires extrapolation of in vitro findings (largely 2D cell culture-based) and animal results into humans. The OOAC simulating human systems can offer a better prediction of draggability, thus reducing the cost of drug development [32]. Third, organ chips can be customized for individual differences (Fig. 2). Based on individual characteristics, researchers can produce individualized organ chips for drug screening studies, which provides more solutions for individual differences in clinical treatment and can better meet the needs of different patients [33]. Last but not least, organ chips can simulate fluid flow in the body (Fig. 2), which is crucial for all cells and tissues in the body. Using OOAC, we can simulate the metabolism of the liver, the purification function of the spleen's blood flow, and the physiological functions of the kidneys, such as reabsorption and transport [34]. In addition, OOAC can be applied to research fields such as rare diseases, stratified medicine, and nanomedicine [35].

The purpose of building organoids and OOAC is to simulate the structure and function of human organs in vitro, in order to better study the pathogenesis of diseases, drug screening evaluation, and toxicological testing. Both of them can be used in drug development to evaluate the effectiveness and safety of drugs. By simulating the response of human organs to drugs, the effects of drugs in humans can be more accurately predicted, reducing the limitations and uncertainties of animal experiments and single-layer cell experiments. However, the two models have some differences in construction, complexity, operability, and some application scenarios. First, organoids are constructed by culturing stem cells or progenitor cells under specific conditions, allowing them to self-assemble to form miniature organ types with three-dimensional structures [36]. For example, intestinal organoids, microscopic organs with intestinal epithelial structure and function, are obtained by culturing intestinal crypt stem cells under specific culture conditions [37]. As a microfluidic chip, OOAC builds a tiny organ model on the chip, and then etches tiny channels and chambers on the chip through microfabrication technology to simulate the physiological environment of human organs [12]. In terms of the complexity of both, organoids are relatively simple compared to organ chips, although they have certain organ structures and functions, their complexity and functional integrity still lag behind that of real organs. OOAC can enable multifaceted simulation of complex organ functions by integrating multiple cell types, biomaterials, and microfluidics, providing more possibilities in simulating real physiological microenvironments and evaluating complex interactions of multi-organs. Secondly, in terms of operability, the culture of organoids is relatively simple and can be carried out on a large scale in the laboratory, mainly by changing the culture conditions to regulate the growth and differentiation of organoids. OOAC requires complex microfabrication technology and equipment support, which is difficult to operate, but it can be precisely controlled by the microfluidic system to adjust the experimental conditions, such as changing fluid velocity, pressure, chemical composition, etc., so as to better simulate the physiological and pathological changes of human organs [38]. Finally, in terms of application scenarios, organoids are often used in basic medical research, such as in organ development, stem cell biology, and disease construction. At the same time, it also has certain application prospects in personalized medicine [39]. For OOAC, because it can accurately simulate the physiological and pathological state of the human body, it has greater advantages in drug screening and toxicology testing. In addition, OOAC can also be used to study organ interactions and systemic diseases, providing a new platform for the study of multi-organ diseases [40].

Overall, the 3D cell culture system, spanning stem cell differentiation from single adult or progenitor cells, integrated spheroids /organoids into OOAC [41]. Organoid and OOAC models are complementary to animal models and 2D cell culture methods, which can better simulate the internal environment of the human body, and they also have great potential in different research fields [15].

## Application of organoids in the study of TCM

Organoids are abundant in various cell types and possess tissue structures that can be harnessed for the development of disease models through the manipulation of culture conditions, genetic editing, and the acquisition of patient-derived cell sources. These models can be instrumental in assessing the effectiveness and mechanism of TCM treatments. Currently, the application of the organoid model in TCM studies has been mainly focused on efficacy and safety evaluation, drug screening, mechanism evaluation and drug interactions.

Organoids have enormous development potential in the TCM studies. However, the corresponding reports are still in limited numbers. Currently, researchers have developed organoids derived from the inner ear [42], skin [43], brain [44], liver [36], intestines [45], kidney [28], and others [46]. In this section, we summarized the current TCM research with the use of different sources of organoids for multiple purposes.

### Intestinal organoids (IOs)

IOs were initially developed in the research laboratory of Hans Clevers, the pioneer of organoid technology, in 2009 [47]. IOs have a complex three-dimensional structure that allows relatively accurate modeling of the cell types and tissue structures that make up the gut [48]. IOs can be obtained either by isolating intestinal crypts from humans or animals or by in vitro differentiation of human ESCs and iPSCs. They are primary cultures that maintain the properties of human intestinal epithelial cells over many generations, and gut organoids can give rise to various types of epithelial cells contained in intestinal crypts or intestinal villus structures [49]. IOs can more realistically mimic the physiopathological state of the human intestinal epithelium, and therefore, they can be used to investigate the therapeutic mechanism of TCM and its ingredients for the treatment of intestinal diseases.

Murine-derived IOs have frequently been applied to evaluate the pharmacological activity and underlying mechanisms of TCM herbs or constituents. The developing method for IOs is mature. Normally, after isolating crypts from the small intestines of mice, crypts are resuspended and seeded into Matrigel using the culture medium of IntestiCult organoid growth medium containing key growth factors. The IOs are readily induced in 3–7 days.

*Glycyrrhizae* radix *et* rhizoma (licorice) is commonly used in various herbal formulas. The C57BL/6J mouse-derived IOs were treated with glycyrrhetinic acid (GA), an active ingredient of licorice. At the same time, immunofluorescent staining of the obtained IOs was then performed and it was found that GA increased the expression of HuR in IOs and significantly promoted the growth and development of isolated IOs [50].

Gegen-Qinlian decoction (GQD) is composed of *Puerariae lobatae* radix, *Scutellariae* radix, *Coptidis* rhizoma, and *Glycyrrhiza* radix *et* rhizoma and is used to treat acute enteritis and bacterial dysentery [51]. One study developed the small intestinal organoids using the methods described above. After researchers treated the IOs with activators of ferroptosis RSL-3 and GQD, the IOs were detected for intestinal permeability, cell viability, mitochondrial reactive oxygen species (ROS), intracellular ferrous ion fluorescence probe, and whole tissue immunofluorescence detection of organoids. And the results showed that GQD was able to inhibit ferroptosis to prevent colonic damage and intestinal epithelial barrier dysfunction [52].

Injured IOs have also been introduced by supplementing proinflammatory factors such as IL-1β and TNFα. Disease modelling can be achieved by developing IOs from disease animals or genetically modified animals. These models are efficient for evaluating efficacy of TCM herbs and constituents as well as their action mechanisms.

For example, Schisandrin C is derived from the *Schisandrae chinensis* fructus. Murine IOs were treated with IL-1β to obtain the injured epithelial model. Treatment by schisandrin C improved IOs permeability by increasing the expression of zonula occludens-1 (ZO-1) and occludins to improve IL-1β-induced intestinal permeability dysfunction, which confirmed the therapeutic effect of schisandrin C on leaky gut [53].

The TCM formula Shenling-baizhu-san has the function of regulating gastrointestinal motility, and it was found that Shenling-baizhu-san could rescue TNFα-mediated pyroptosis of murine IOs [54]. Additionally, rosavin, an active constituent of *Rhodiola*, was recently found to alleviate TNFα-mediated injury of murine IOs [55].

Proanthocyanidins are natural plant components with strong antioxidant activity and have anti-tumor, preventive, and therapeutic effects on cardiovascular diseases [56]. In one study, the therapeutic effects of procyanidin B2 on intestinal diseases were investigated by isolating mouse intestinal crypt cells for establishing Nrf2 knocking-down IOs, and the results showed that procyanidin B2 could reduce the occurrence of colitis-associated neoplasms by decreasing the accumulation of ROS, protecting the intestinal tract from radiation, and promoting the repair of intestinal damage [57].

### Liver organoids

Liver-like organ disease models generated from ASCs or iPSCs enable the screening of drugs with therapeutic effects on liver disease as well as toxicological tests [36]. Hepatocytes in liver organoids are structurally and functionally highly similar to the internal human liver, and their metabolic work can mimic the efficacy and toxicity of drugs in the human body in a high-throughput manner. Liver organoids can also mimic the individualization of human liver pathology, providing a viable option for personalized treatment of liver disease [58]. The long-term culture of mature human hepatocytes is essential for the development of toxicological in vitro research methods and the study of hepatotropic infections, as well as the study of various hereditary and metabolic liver diseases. The 3D culture system of the liver in vitro can achieve long-term expansion of primary mouse and human hepatocytes, which is beneficial to the study of liver diseases [59]. At the same time, this also provides new ideas for the screening and evaluation of natural active ingredients with therapeutic effects on liver disease in TCM.

For example, Liu et al. investigated the hepatotoxicity of 2,3,5,4′-tetrahydroxy-*trans*-stilbene-2-o-*β*-glucoside (*trans*-SG) and its *cis* isomer (*cis*-SG) [60]. They found that *cis*-SG showed higher hepatotoxicity in LPS-treated rat model than *trans*-SG [60]. However, in the 2D hepatic cell model, *cis*-SG and *trans*-SG did not show the difference in hepatotoxicity [60]. Finally, in vitro 3D liver organoid models were constructed by culturing human hepatoma cell lines and HepaRG cells in complete William's Media E. The resulting liver organoid models were treated with corresponding drugs for cell viability detection, high-content imaging analysis, real-time fluorescence quantitative detection, and flow cytometry analysis. Similar results were obtained with that of the in vivo experiment. Moreover, the hepatocytotoxicity of *cis*-SG was found to be mainly due to its damage to cellular mitochondria in liver organoids [60].

**Fig. 3:**
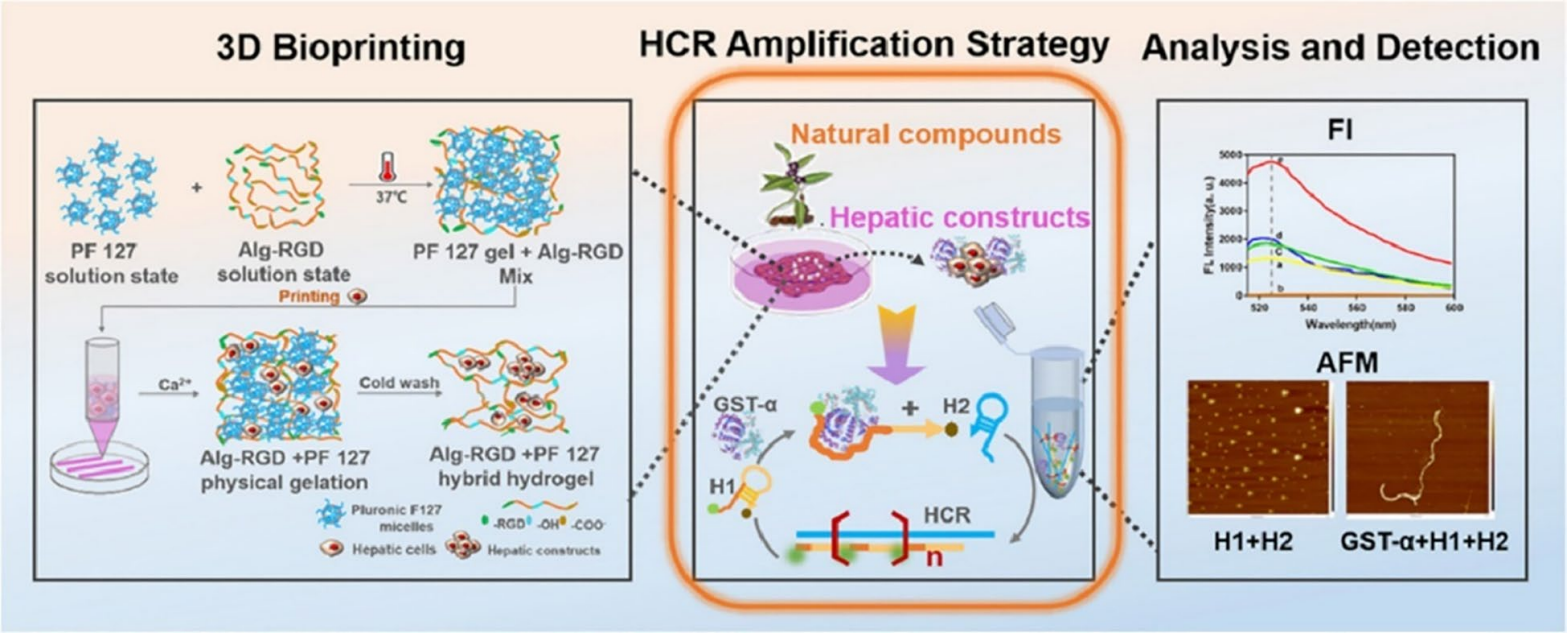
The 3D hepar-on-a-sensor-platform with hybridization chain reaction (HCR) amplification strategy to intuitively assess the hepatoxicity of natural compounds. The system was integrated with a powerful fluorescent sensor based on the HCR amplification strategy, aiming to monitor the early hepatotoxic biomarker, namely glutathione-S-transferase-alpha (GST-α) that is secreted by the 3D liver construct. Reprinted with permission from ref. [61]. Copyright 2023 Acta Materialia Inc. Published by Elsevier Ltd

Qian et al. recently successfully constructed a 3D liver model in vitro by depositing heat-responsive alginate-RGD/Pluronic hydrogel, which was used to evaluate the liver toxicity of two known hepatotoxic natural compounds, emodin and triptolide (Fig. 3) [61]. The system was integrated with a powerful fluorescent sensor based on the hybridization chain reaction amplification strategy (HCR), aiming to monitor the early hepatotoxic biomarker, namely glutathione-S-transferase-alpha (GST-α) that is secreted by the 3D liver construct. This study accurately evaluates the liver toxicity of natural compounds in vitro, which also provides a new way for the toxicity assessment of TCM.

### Kidney organoids

The kidney consists of a variety of cells and tissues and has the functions of maintaining the stability of the internal environment, removing nitrogen wastes, regulating blood pressure, bone density, etc. [62]. The renal structure and function are complex and susceptible to the influence of external factors. The complex structure and function of the kidneys make them susceptible to external factors, such as chronic kidney disease (CKD), which affects 13% of the world's population, including polycystic kidney disease (PKD), alport syndrome, cystinosis, fabry disease, nodular sclerosis, gitelman syndrome, and other diseases [63]. The kidneys are responsible for the excretion of several substances from the body. The kidney removes some drugs from the body through excretory action, while some drugs tend to be nephrotoxic. Renal organoids can mimic the nephrotoxicity of drugs in vitro. Compared with 2D cell culture, renal organoids have the following advantages: firstly, they contain multiple cell types, and the action of drugs on the kidney is often dependent on multiple cells, so renal organoids can better match the action of drugs in vivo; secondly, organoids can be cultured for a longer period, and they can retain the specific phenotypes of certain cell types [64]. These characteristics of renal organoids make it a powerful tool for the development and screening of active ingredients in TCM. For example, esculentoside A derived from *Phytolaccae* radix has significant nephrotoxicity, and its toxicity was recently evaluated by human kidney organoids derived from iPSCs. It was found that the toxicity of *Phytolaccae* radix was related to the epithelial-mesenchymal transition of STING signaling [65].

### Lung organoids

The lung has a complex structural composition and function. Lung-derived organoids can be classified according to their origin: airway basal cell-like organoid, airway secretory cell-like organoid, alveolar type II epithelial cell-like organoid, distal airway pluripotent progenitor cell-like organoid, and human pluripotent stem cells (hPSCs)-like organoid [66]. Organoids derived from lung ASCs can be used to mimic the interaction between stem cells and their microenvironment [67]. Lung organoids have become an effective tool for modeling the physiological and pathological conditions of the lung and have been used for drug development and screening evaluation.

It was found that the natural flavonoid luteolin enhanced trans-epithelial sodium transport in alveolar models. The researchers isolated AT2 cells from adult mouse lungs to culture alveolar epithelial organoids in vitro, and then used confocal microscopy to identify the resulting organoids. After treating the mouse alveolar epithelial organoids with luteolin, they found that luteolin was able to reverse the LPS-decreased epithelial sodium channel expression at both protein and transcriptional levels [68].

### Brain-like organoids

Brain-like organs contain a variety of cell types with neuronal characteristics and functions similar to those of in vivo brain tissue, and are more representative of in vivo physiology than 2D culture [69]. Organoids that contain multiple regions of the brain can be referred to as brain organoids, while organoids that resemble specific regions of the brain can be referred to as organoids of that region [70], for example, "striatal organoids" [71]. Brain organoids can be generated from human-derived iPSCs or ESCs, capable of mimicking aspects of embryonic cortical development, and can also be used to study neurological disorders such as microcephaly and autism [72].

Brain organoids, also known as the human miniature brain (MB), can mimic the complexity of the human brain in vitro. In one study, the researchers developed cerebral organoids based on the previously reported feeder-dependent iPSCs method [73]. The obtained brain organoids were treated with a 4.7-kDa ginseng polysaccharide. It was demonstrated that a 4.7-kDa ginseng polysaccharide could reduce Aβ aggregation and neuroinflammation in cerebral organoids [74].

In many studies, rotenone is commonly used in modeling neurological diseases such as Parkinson's disease [75–78]. In order to investigate the effect of *codonopsis pilosula* polysaccharide on the neuronal toxicity induced by rotenone, the researchers used the NE-4C embryonic neuroectodermal stem cells for establishing brain organoids, which included several rounds of culture and supplementation of specific inducing medium [15]. After a 10-day culture, the mouse brain organoids formed. The obtained brain organoids were treated with rotenone and *codonopsis pilosula* polysaccharide. It was found that *codonopsis pilosula* polysaccharide could improve the cytotoxicity and DNA methylation of rotenone on mouse brain tissues and organs, and increase intracellular glutathione activity. It was also found that Ginseng polysaccharides could ameliorate the cytotoxicity and DNA methylation of rotenone on mouse brain organoids and increase intracellular glutathione activity [15].

### Tumor organoids

TCM has been used in cancer treatment for a long time. It can not only relieve the symptoms of cancer patients, but also control the size of tumors and prolong the survival time of cancer patients [79]. For various types of cancer, research is increasingly focusing on personalized therapies with natural products including the TCM source [80]. Tumor-like tumors mimic the primary tumor both structurally and functionally, and retain histopathological, genetic, and even therapeutic responses that are highly similar to the primary tumor. Compared with patient-derived xenograft (PDX) models, tumor-like tumors require less time and tissue to construct, and can stabilize key characteristics of the primary tumor even after long-term proliferation [81]. Patient-derived cancer organoids, which retain heterogeneity of tumors, offer an efficient model for drug screening and evaluation [21].

Organoid models have been used to simulate cancer initiation, metastasis, and drug treatment response in the most common gastrointestinal cancers [82]. The bark and pericarp of *Ailanthus altissima* can be used as medicine, and modern studies have proved that it also has certain therapeutic effects on gastric cancer. Ailanthone (AIL) extracted from *Ailanthus altissima* could down-regulate the expression of p23 to induce the inhibition of base excision repair, thus promoting the apoptosis of gastric cancer cells. The effect of AIL on the spheroidization ability of patient-derived gastric cancer-like organs was evaluated. The results showed that AIL effectively inhibited the spheroidization ability of gastric cancer-like organs, suggesting that AIL has a good anticancer effect [83].

Pien Tze Huang has the efficacy of clearing heat and removing toxins, subduing swelling, and relieving pain. Studies have shown that Pien Tze Huang also inhibited the occurrence of colon cancer [84]. The ginsenosides contained in Pien Tze Huang are sourced from the rhizomes of *Panax ginseng*, *Panax notoginseng* and *Cinnamomum cassia* [85]. Researchers constructed an organoid model of colorectal cancer patient origin to screen the active ingredients in Pien Tze Huang, and the results showed that ginsenoside F2 and ginsenoside Re contained in Pien Tze Huang have inhibitory effects on the growth of the organoid model of colorectal cancer patient origin [84].

Decursin is a coumarin-like compound extracted from *Angelicae sinensis* radix and other herbs, and in order to understand the anti-tumor mechanism of decursin, Solbi Kim et al*.* established a gastric cancer patient-derived cancer organoid model [86]. They found that decursin could inhibit the growth of the patient-derived gastric tumor organoids by decreasing the autophagy disorder induced by lysosomal protein cathepsin C and thus inhibiting the growth of gastric cancer organoids [86].

Halofuginone in *Dichroae* radix has the effect of treating malaria, and the combined effect of halofuginone and cisplatin was investigated. The researchers cut surgical samples or bronchial biopsy tissue from lung cancer patients into small pieces and digested them with collagenase. After the digestion was completed, they were washed and centrifuged with fresh medium again. The dissociated cells were seeded and cultured to obtain human lung cancer organoids. After treating them with cisplatin and halofuginone, the synergy effect between the drugs was observed. It was found that the antitumor activity of halofuginone and cisplatin was significantly enhanced [87].

The mortality rate of liver cancer is high, and TCM represents a good screening platform for the identification of new treatments [88]. By using human liver tumor organoids, it was found that a naturally occurring compound oroxylin A could directly target transketolase to inhibit the non-oxidative phosphonate-pentose pathway and activate p53 signaling to inhibit the growth of patient-derived hepatocellular carcinoma tumor-like organs [89].

Ainsliadimer, a natural product extracted from *Ainsliaea glabra*, is a new guaiacolide sesquiterpene dimer, and a colorectal cancer-derived organoid model was constructed in the study to screen natural drug components with anti-colorectal cancer effects. It was found that ainsliadimer was able to inhibit the growth of colorectal cancer cells and colorectal cancer tumor organoids [90].

Taubenschmid-Stowers et al*.* developed cerebral organoids using human ESCs [91]. After oncogene amplification using sleeping beauty (SB) transposase or the use of CRISPR-Cas9 system to induce tumor suppressor gene mutations to induce tumorigenesis, two brain tumor organoids were generated: one was a central nervous system primitive neuroectodermal tumor (CNS-PNET) induced by c-MYC overexpression, and the other was a glioblastoma-like tumor group-2 (GBM-2) induced by suppressor genes p53, NF1 and PTEN. After the obtained brain tumors were treated with dihydroartemisinin and 5-aminolevulinic acid (5-ALA), the nerve cell status of the tumor organoids was monitored and sub-analyzed by flow cytometry and immunohistochemical staining. It was found that the combination of 5-ALA and dihydroartemisinin significantly reduced the number of tumor cells in the central nervous system primitive neuroectodermal tumor (CNS-PNET-like) neoplasm model, and provided elevated ROS levels and increased muscle DNA damage to increase tumor cell death, exerting a powerful anti-cancer effect [91].

### Other sources

Rheumatoid arthritis is a chronic and complex autoimmune disease. The disease is highly heterogeneous, and there are differences in the genotype, disease course and symptom manifestations of the disease. The therapeutic drugs on the market also make it difficult to achieve desired therapeutic effect [92]. Sesame seeds contain some functional components, such as sesamol, which has a positive effect on human health and acts as an antioxidant, anticancer, protects against neuronal damage, and is anti-inflammatory [93]. The researchers used trypsin to isolate rheumatoid arthritis fibroblast-like synoviocytes (RA-FLS), resuspend them in a cold Matrigel basement membrane for a period of time, and then place them in complete medium for a period of time to obtain synovial organoids. After the sesamol treatment, it was demonstrated that sesamol could delay the progression of rheumatoid arthritis and also inhibit the growth of synovial organoids [94].

Pancreatic cancer is one of the most lethal malignancies, and there is an urgent need to build effective models to explore its pathogenesis and therapeutic drugs. Pancreatic organoids can be rapidly generated from resected tumors and biopsies, and they can be preserved under cryopreservation conditions. They can exhibit ductal and disease-stage heterogeneity [95]. Gemcitabine (GEM) is commonly used in the treatment of pancreatic ductal carcinoma, but long-term use of GEM will produce drug resistance. Researchers investigated the mechanism and effect of the combination of andrographis and GEM by constructing gemcitabine-resistant pancreatic ductal adenocarcinoma cells and patient-derived organoid models. The results showed that the combination of andrographis and GEM not only enhanced the therapeutic effect of gemcitabine, but also reversed the drug resistance [96].

Cardiac organoids are mostly 3D structures formed spontaneously by self-assembling aggregates of iPSCs on the anti-adhesion surface. They contain the major cell types of the heart, including cardiomyocytes, cardiac fibroblasts, and endothelial cells [97]. Guanxinning injection is used to treat angina pectoris in coronary artery disease, but the mechanism of its treatment of heart failure is still unclear. In order to evaluate its therapeutic effect on heart failure, one study utilized primary cardiac fibroblasts, cardiomyocytes and endothelial cells obtained from neonatal Sprague–Dawley rats to obtain a 3D cardiac organ-like model and a transverse aortic constriction (TAC) model [98]. The organoid model of myocardial hypertrophy was treated by guanxinning injection and metoprolol respectively. It was found that guanxinning injection could improve deoxyepinephrine-induced myocardial hypertrophy, apoptosis, and mitochondrial dysfunction [98] and thus has a therapeutic effect on heart failure.

## Co-culture of organoids with other type of cells

Organoids are a high-quality platform for simulating the function and physiological state of human organs in vitro, but these organoid culture models lack interaction with other relevant cells in the microenvironment. If organoids can be co-cultured with other cells, then a simulation platform closer to the in vivo microenvironment will be obtained, which will greatly improve the precision of in vitro drug screening and evaluation. Thus, we will further describe the co-culture system of organoids with different types of cells.

**Fig. 4:**
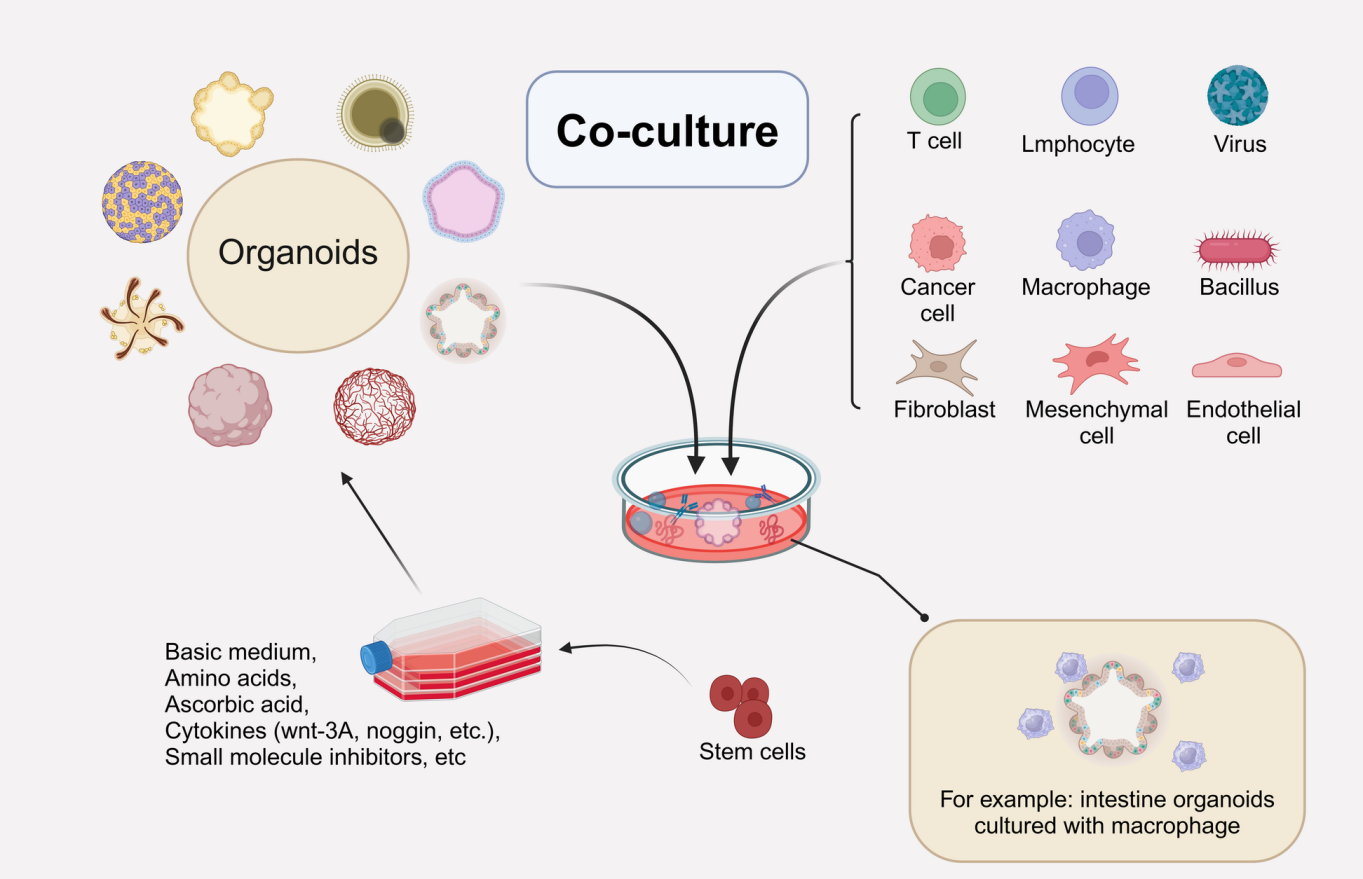
To study the interaction with other relevant cells in the microenvironment, organoids can be co-cultured with various immune cells, cancer cells, microorganisms (bacteria, fungi, viruses), etc. For example, the co-culture system with macrophages provides intestinal organoids with an intestinal immune microenvironment similar to that within in vivo

Organoid technology, as an in vitro culture system simulating human organs, can mimic the complex environment in the human body and further explore the therapeutic effects and toxic reactions of drugs; however, organoids still lack interactions with different types of cells and microorganisms (viruses, bacteria, etc*.*). For example, in tumor therapy research, the development of tumors involves complex interactions between malignant transformed cells and the tumor microenvironment (TME), and co-culture can provide this complex microenvironment for tumor cells [99]. Therefore, if organoids are co-cultured with other different cells and microorganisms, they can simulate the physiological microenvironment in vivo, as well as diseases such as bacterial or viral infections (Fig. 4). For instance, many bacteria can act inside tumors and produce therapeutic chemicals, and in order to further explore the mechanism of action, it is necessary to construct a bacterial spherical co-culture system in vitro [100]. This multi-cellular and multi-microbial co-culture system in a complex environment can be used to better evaluate the efficacy and toxicity of the drugs, and thus to more efficiently and accurately screen the drugs and the natural active ingredients of the drugs that have the potential for clinical application.

Valtrate is a natural product derived from *Valeriana officinalis* L. In one study, the researchers constructed a co-culture system of glioblastoma spheroids with a brain organoid model to investigate its anti-tumor mechanism, and the experimental results showed that after treatment of the co-culture system with valtrate, the number of GBM cells invading brain organoid cells was significantly reduced and the area of glioblastoma cell invasion into the brain organoid model was significantly reduced [101].

Another study established a co-culture system of immune cells and IOs. It was found that high levels of ROS were observed in the untreated co-culture model, and immune cells and peritoneal macrophages were found to be highly reactive. The co-culture system of immune cells and intestinal organoids provides IOs with an intestinal immune microenvironment similar to that in vivo, and the results of this experiment also suggest that Changyanning formula controlled ROS production by modulating the intestinal immune microenvironment, thus exerting a therapeutic effect on enteritis [102].

As a TCM herb, *Ephedrae herba* can be used to treat coughs, colds, and other diseases. Studies have shown that ephedra could inhibit norovirus infection in stem cell-derived human IOs [103].

Other researchers constructed a co-culture system composed of IOs and lamina propria lymphocytes (LPLs) to explore the repair and therapeutic effect of *dendrobium fimbriatum* Hook polysaccharide on intestinal epithelium damaged by sodium sulfate [79], and it was found that *dendrobium fimbriatum* polysaccharides could promote the regeneration of intestinal stem cells to protect intestinal mucosal integrity and improve ulcerative enteritis through LPLs-mediated IL-22 upregulation [104].

Co-culture techniques can well mimic the interactions between a wide range of cells, between cells and microbes, and between cells and the immune microenvironment, but they cannot fully mimic secretion and metabolism in vivo. In vivo, more complex paracrine signaling, autologous extracellular matrix, and angiogenesis in cancer-associated fibroblasts [105] still limit the application of organoid co-culture techniques in drug screening.

## Application of OOAC in medical and TCM research

In the process of drug research and development, new technologies are emerging to speed up the development of new drugs and reduce the cost of research and development. OOAC are devices that combine microfluidics and cell culture to simulate pathophysiology at the tissue and organ level in vivo, and microfluidics allows for continuous nutrient exchange, better oxygen perfusion, and physiological shear stress compared to static in vitro culture models [34], which better mimic physiological conditions in vivo. Moreover, OOAC could help build multi-organ systems, which enables the study of multi-system interactions. Researchers can analyze the course of the disease and the therapeutic effect of drugs on the disease by controlling the local cellular, molecular, chemical and other parameters of the organ chip [106]. Huh et al. also describe the characteristics of microfluidic technology in three aspects: easier to control biomedical microenvironment, multi-functional construction of multi-organ systems, and lower viability during parallel experiments [107]. In this regard, OOAC plays an important role in the screening and therapeutic evaluation of active ingredients in TCM, clarifying the compatibility between TCM herbs, and the toxicological and pharmacological mechanisms of TCM [1].

OOAC can be used in disease research to explore pathological mechanisms. Organ chips can simulate the occurrence and development of various diseases in vitro, helping researchers gain a deeper understanding of the pathological mechanisms of diseases. For example, using lung chips to simulate pathological processes such as lung diseases and asthma, it is possible to observe cell changes, verify responses, and abnormal gas exchange functions [108].

Microfluidic channels in organ chips allow for the exchange of compounds and other signals in tissues [109], which allows organ chips to be used for drug toxicity evaluation. OOAC has been widely used in various toxicity tests for cosmetics [110], food additives [111], environment [112] and drugs [113]. Currently, the liver, kidney, heart, nerve, intestines, lungs, blood-brain barrier, and some multi-organ chips (liver and kidney) have been used for drug toxicity evaluation [114]. For instance, in order to explore the toxicity of nitidine chloride, chelerythrine chloride, magnoflorine chloride, and hesperidin on cardiomyocytes, the researchers constructed an in vitro model of the heart using OOAC technology and found that the four compounds had little effect on cardiomyocyte activity at concentrations below 10 μmol/L [115]. *Mentha spicaia* L. has antipyretic, antitussive, anti-infective, and anti-inflammatory effects, etc. Researchers constructed a kidney chip as an in vitro model to investigate the nephrotoxicity of kaempferol, a naturally occurring compound. The kidney chip is able to mimic the process of substance exchange in the human kidney, and the morphology of the human embryonic renal cells in the kidney chip can be directly observed under an optical microscope. The results showed that treatment with 30 μM kaempferol did not cause damage or apoptosis of human embryonic renal cells [116]. Consequently, organ chips can be used to predict the potential toxicity of drugs before they are marketed. By simulating drug use scenarios on organ chips, observing the impact of drugs on various organs, early detection and prediction of drug adverse reactions can reduce the risk of clinical use of drugs [109].

**Fig. 5:**
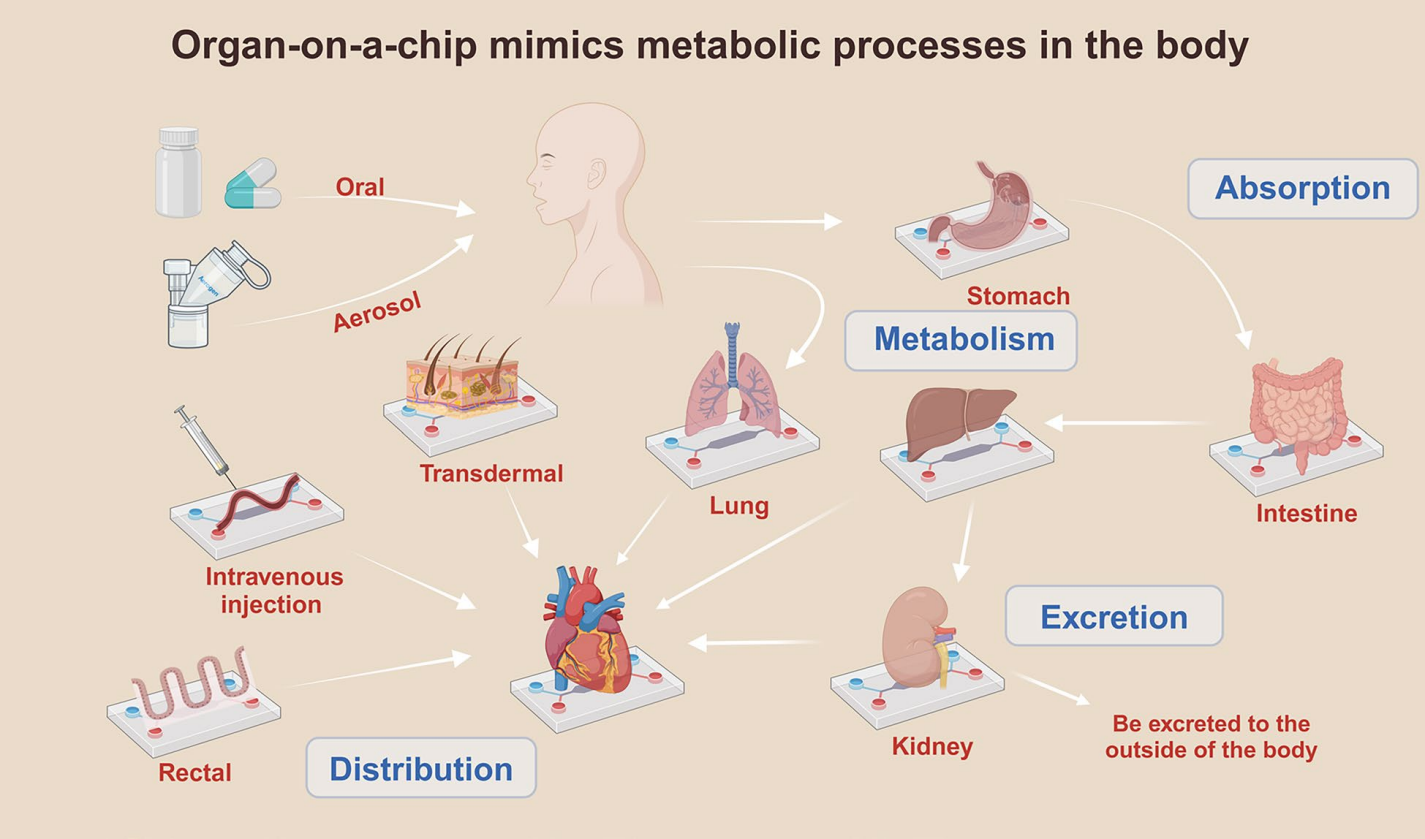
Various organ-on-a-chip connection applications can simulate the drug delivery metabolic system in vivo relatively and completely in vitro. In this framework, multiple organs are involved including lungs, liver, blood vessels, intestines, heart, kidneys, stomach, and transdermal tissues

Moreover, the multi-organ-on-chip (MOC) model can connect isolated organs through microfluidic channels to create an ideal pharmacokinetic and pharmacodynamic (PK-PD) model for detecting complex interactions between multiple organs and the co-production of multiple organ responses to drugs [117] (Fig. 5). In this framework, multiple organs are involved including lungs, liver, blood vessels, intestines, heart, kidneys, stomach, and transdermal tissues. The model is able to simulate key drug processes of absorption, distribution, metabolism and excretion, providing an approach for monitoring the dynamic responses and disposition of multiple organs to drugs. Specifically, MOC can be used to simulate the transformation of a drug in vivo into a metabolite that acts on another tissue, to simulate the toxicity of the compound in non-target tissues, and to elucidate the quantitative effect of tissue on the bioavailability of a drug in an intended target tissue [118]. MOC can also connect various OOAC models to predict the optimal efficacy of various drug prescriptions in target organs, while minimizing the toxicity of drugs to other organs [72]. However, so far, no MOC has been found used for the study of TCM.

OOAC is also used in the field of personalized therapy, where patient-specific OOAC models are constructed. Based on individual patient differences, it is able to evaluate patient response to drugs and the therapeutic effect of drugs. Organ chips made from patient-derived cells could be used to simulate rare genetic diseases. When creating multiple chips, the cells within each chip can be derived from different donors in patients with different comorbidities, designing and optimizing drugs for specific combination diseases, minimizing toxicity, determining optimal drug delivery routes, and predicting PK-PD in patients [119]. In addition, it can also combine gene editing technology to develop the effectiveness and safety of new treatment methods for diseases on organ chips, providing a reference for the clinical application of new treatment methods for diseases [120].

Overall, the application of OOAC in the exploration of active ingredients in TCM is currently less reported. As an emerging technology, OOAC needs to be further developed and improved in drug discovery, especially in the study of TCM.

## Current challenges and future prospects

**Fig. 6:**
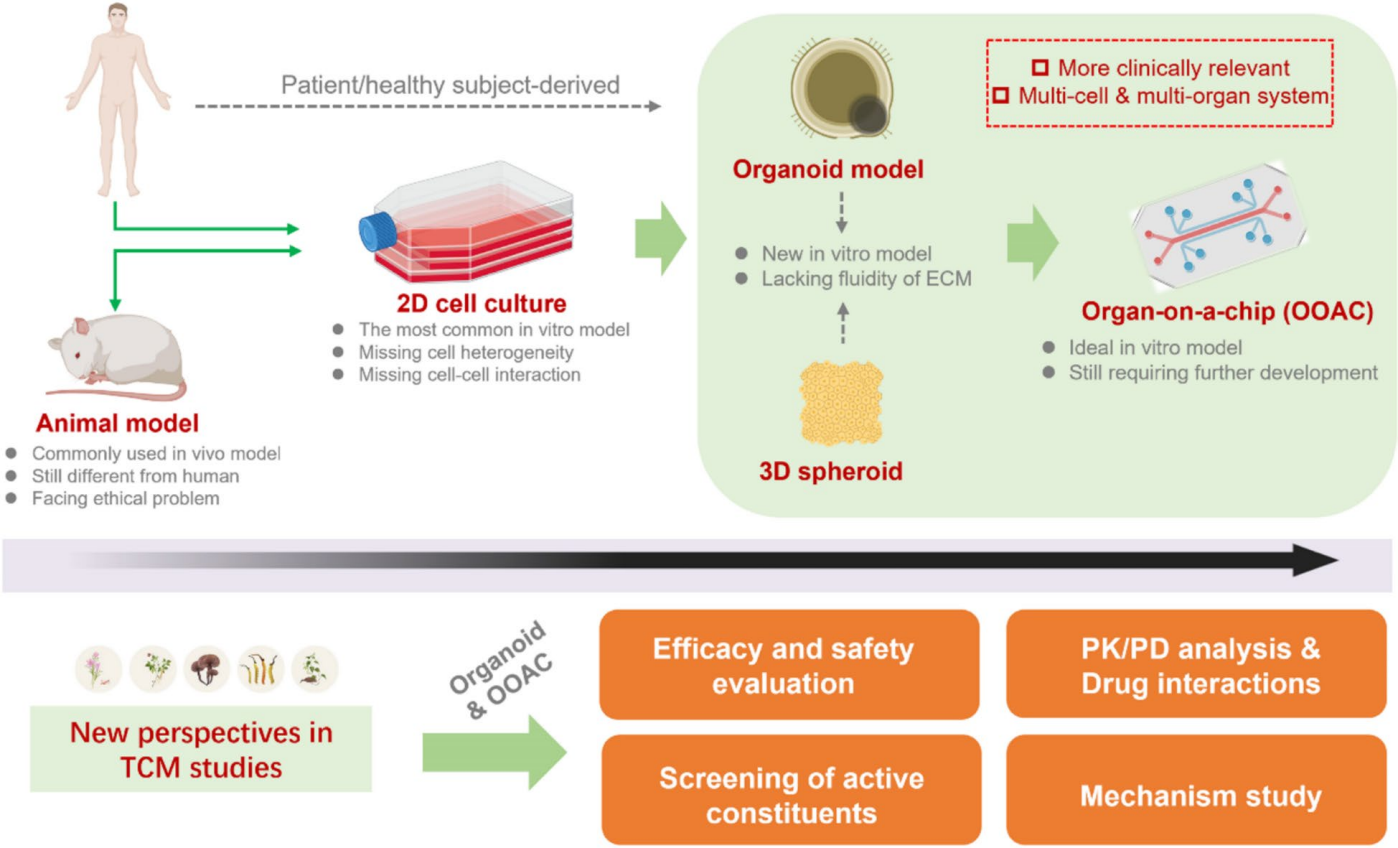
From monolayer cell culture and animal models to organoid technologies and organ-on-a-chip (OOAC) models, technologies for drug screening and evaluation continue to evolve. The emerging organoid and OOAC technologies effectively make up for some of the shortcomings of older models. Generally, the organoids and OOAC, containing cell heterogeneity and cell-to-cell communication or organ-to-organ interaction, could be more clinically relevant, which facilitates the TCM research in the field of efficacy/safety evaluation, PK-PD analysis, drug interactions, screening of active constituents and mechanism study

The 2D monolayer cell culture method is easy to set up and is most widely used in drug screening, but it lacks cell-to-cell and cell-microenvironment interactions (Fig. 6). 3D cell models can better simulate the effects of drugs in vivo. Compared to standard 2D cell culture models, 3D cell models are more predictive of gene and protein expression, metabolic function, and physiological function [121]. However, the 3D culture system lacks the stressful effects of fluids and tissues in the body's environment on the cells and does not reproduce the supply of nutrients, inorganic salts, and other substances to the cells from the bloodstream [122]. Organoids can provide sufficient nutrients and oxygen for cultured cells, and can reproduce the 3D growth structure and interactions between multiple cells; the matrix in organoids is in a flow state, which can better simulate cell metabolism in vivo, effectively preventing metabolic substances from accumulating in cell tissues and releasing toxic substances over time (Fig. 6). It should be noted that organoids can be co-cultured with other cell types which is beneficial for investigating multi-systems interactions. Moreover, organoids derived from a tissue contain sufficiently more cell heterogeneity than both 2D and 3D cultures. They can be derived from human sources. Therefore, organoids simulating a specific tissue or organ can be more clinically relevant than other conventional cell models. At present, both brain organoids and retinal organoids have been studied to demonstrate their ability to simulate the development of human organs, which cannot be observed in animal models [123]. This is also an advantage of organoid models over animal models. Due to the difficulty of conducting safe clinical trials of many drugs in the elderly, children and pregnant women, researchers were able to use organ chips to study the effects of drugs and various chemical components on the elderly and children, as well as the ability of drugs to pass through the placental barrier [119]. In addition, in recent years, extracellular vesicles (EVs) have become a hotspot in biomedical research due to their nano-scale structure, low immunogenicity, good biocompatibility and drug delivery capabilities. Organoid extracellular vesicles (OEVs) have also received extensive attention. Compared with traditional EVs, OEVs are more abundant and have better biological characteristics and therapeutic effects. OEVs can be used for liquid biopsies, pharmacological testing, toxicity testing, disease treatment, customized personalized medicine, genetic research, immune regulation, and epithelial repair, and OEVs may become a more promising treatment for bone diseases [124].

The OACC contains the internal fluid flow within organoids, which can better simulate the tissue or organ function. Moreover, it can simulate long-term in vitro drug experiments and explore the effects of long-term drug use on cells, tissues, and organs [7] (Fig. 6). Most importantly, the use of MOC which consists of multiple organs provides many possibilities for medical research. When the organs are derived from human sources, the MOC can simulate the human system. As a result, the model can be more clinically predictive (Fig. 6). The MOC system can be optimized based on different research purposes. Therefore, the OACC and MOC exhibit certain advantages over animal studies, although they are still less systematic and procedural than animal models [25].

However, organoids and OOAC are still in the developmental stage, and there are still some obstacles to their application. In this part, we will discuss the current challenges and future prospects regarding the following two aspects: the technique side and the application in TCM research.

### The improvement of organoids and OOAC techniques

Organoids and OOAC possess limitations such as the limited lifespan of the cells used in organoids and the insufficient function of drug transporters and luminal metabolizing enzymes in organoids for drug toxicology studies [125]. The current OOAC models cannot fully reproduce the real functions of organs in vivo.

For organoids, the precision of their structure and function needs to be improved. Most organoids currently lack a complete vascular system, which is essential for the organ's nutrient supply, oxygen delivery, and elimination of metabolic waste. Lack of blood vessels can lead to an insufficient supply of nutrients to the cells inside the organoids, affecting their long-term survival and normal function, especially for organoids with high oxygen and nutrient requirements, such as the liver, heart, etc. In the future, it is necessary to explore more efficient ways to construct the vascular system of organoids, such as using vascular endothelial cells to co-culture with organoid cells [126]. To enhance the physiological authenticity of organoids, an understanding of cellular composition and complexity of organoids is also required. Real human organs are composed of a variety of different types of cells, which have specific distributions and interactions in spatial structure. In most cases, current organoids are relatively simple in cellular composition and structure, making it difficult to fully replicate the complexity of real organs. The 3D model represents a simplified organ model, which does not fully replicate the large amount of cellular heterogeneity in organs in the body, and lacks the complex structure of vascular cells and organs [127]. Organoids can be cultured in vitro for a long time, but with the extension of culture time, organoids will also have problems such as cell aging, gene mutation, and functional degradation, which have a certain impact on the application of organoids in long-term research. Therefore, it is also necessary to improve the stability of the long-term culture of organoids to maintain the cell viability and function of organoids. For decades, researchers have made tremendous breakthroughs in the technology of organoid construction, and the same batch of organoids has shown great similarities in all aspects, but the organoids produced by different culture protocols will still be different. Therefore, a multi-faceted evaluation of the constructed organoid model is required, for example, multi-omics analysis, single-cell analysis, spatial analysis, pathological morphological analysis, and functional property analysis are used to evaluate whether organoids can more accurately reflect the spatial structure and function, gene expression, and development of tissue cells in human organs [128]. Additionally, at present, there is still a lack of tools for real-time monitoring of organoids. The lack of real-time monitoring of organoids hinders the evaluation of disease progression and drug efficacy. Some studies have effectively solved this problem by constructing hepar-on-a-chip embedded with graphene quantum dot-capped gold nanoparticle-based plasmonic sensors. Such sensors have more powerful surface-enhanced Raman scattering (SERS), biological stability, and signal consistency, which also provides a better platform for liver injury monitoring [129].

In terms of the development of organ chips, the stability of the accuracy of organ chips should be continuously improved, so that they are closer to the physiological and pathological states of real organs in the human body. For example, further optimizing microfluidic technology to precisely control fluid parameters and improve the stability of the cell growth environment. At the same time, the technological innovation of fusion of organ-like and multi-chip technologies is strengthened to improve the accuracy of simulating real organ functions. Secondly, to expand the application field of organ chips, it is of help to use their multi-organ chips to jointly become body-on-chip to further realize the research on the multi-target mechanism of TCM. It is thus advocated that enterprises increase investment in the research and development of organ chip technology, promote the industrialization of organ chip technology, establish unified industry standards and norms, and ensure the quality and safety of organ chip products. Overall, as an innovative technology in the medical field, organ chip technology has great potential for development. Through continuous technological innovation and application expansion, organ chip technology should make greater contributions to human health.

### The application of organoids and OOAC in TCM research

The chemical compositions of TCM are complex, which inevitably leads to multiple targets of TCM. There may be insoluble active ingredients in herbal preparations and some pharmacologically active metabolites produced by systemic metabolism [130]. This poses a great difficulty in the research and development of TCM and the identification of active ingredients. The pharmacokinetics and metabolism of TCM greatly affect the efficacy and toxicity. Most drugs are absorbed and metabolized by the intestine and liver before reaching other organs in the body to produce their effects, a process that is difficult to achieve by establishing a single organ model in vitro, and it is difficult to simulate the metabolic process of TCM in the human body only by multiple single culture models in vitro. The nature of organoids and OOAC, multi-cell and multi-organ features, makes them suitable for TCM research in several areas.

Firstly, organoids and OOAC provide an excellent model for PK-PD analysis of TCM and drug-drug interactions. Co-culture of organoids with different cell types, or the design of MOC devices will greatly benefit the study of the complex pharmacokinetics and metabolism processes of TCM constituents within a complete system. This further aids the evaluation of dynamic pharmacological effect of TCM. The study of drug-drug interaction, as well as herb-drug interaction, will also benefit from organoids and OOAC models.

Secondly, identification of active constituents of TCM will be more reliable. The PK-PD analysis of TCM using organoids and OOAC models facilitates comprehensive understanding of specific active constituents. On the other hand, organoids and OOAC models can be clinically relevant, so the discovery of active constituents of TCM will be more efficient.

Thirdly, organoids and OOAC models facilitate efficacy and safety evaluation of TCM essentially, as they take into account the compound metabolisms and cell-to-cell or organ-to-organ interactions. Human-derived organoids and OOAC also help to make the results more clinically relevant. Notably, with the help of other modern techniques like omics study, the exploration of action mechanisms of TCM, as well as the discovery of molecular targets, will become more comprehensive.

Therefore, in future research on TCM, more attention should be paid to multi-cell and multi-organ co-culture, integrating multiple organ chips to achieve a complete simulation of the drug metabolism system in vivo, so as to improve the accuracy and reproducibility of organ chips in TCM research.

The combination of traditional advantages of TCM and modern technology is an important direction in promoting TCM to the whole world, and in this process, the application of emerging technologies such as organoid as well as OACC effectively promotes the modernization of TCM. In particular, the continuous development of organ chips can make up for some of shortcomings of 2D and 3D cell culture, which also brings new opportunities for the development of TCM (Fig. 6). Generally, the organoids and OOAC, containing cell heterogeneity and cell-to-cell communication or organ-to-organ interaction, could be more clinically relevant, which facilitates the TCM research in the field of efficacy/safety evaluation, PK-PD analysis, drug interactions, screening of active constituents and mechanism study. These new technologies not only provide new research methods for TCM research but also provide technical support for the transformation and advancement of TCM modernization.

## Conclusion

In this review, we comprehensively summarized organoids and OACC technology in the field of TCM research. At present, due to technical limitations, organoids and OACC technology have not been widely used in the screening of active ingredients and toxicology research of TCM. OACC increases the fluidity on the basis of organoids, which can be better used in PK-PD research of TCM, so as to ensure the safety and effectiveness of drugs. In the future, through the further development of organoid and OACC technology, we hope to have a deeper understanding and further development of TCM.

## Data Availability

Not Applicable.

## Abbreviations

OOAC: Organ-on-a-chip
TCM: Traditional Chinese medicine
3D: Three-dimension
2D: Two-dimension
iPSCs: Induced pluripotent stem cells
ESCs: Embryonic stem cells
ASCs: Adult stem cells
PDMS: Polymer dimethylsiloxane
BM-MSCs: Bone marrow-derived mesenchymal stem cells
Ios: Intestinal organoids
GA: Glycyrrhetinic acid
GQD: Gegen-Qinlian decotion
ZO-1: Zonula occludens-1
ROS: Reactive oxygen species
CKD: Chronic kidney disease
PKD: Polycystic kidney disease
hPSCs: Human pluripotent stem cells
MB: Miniature brain
PDX: Patient-derived xenograft
AIL: Ailanthone
5-ALA: 5-Aminolevulinic acid
CNS-PNET-like: Central nervous system primitive neuroectodermal tumor
RA-FLS: Rheumatoid arthritis fibroblast-like synoviocytes
GEM: Gemcitabine
TAC: Transverse aortic construction
TME: Tumor microenvironment
LPLs: Lamina propria lymphocytes
DSS: Dextran sulfate sodium
MOC: Multi-organ-on-chip
PK-PD: Pharmacokinetic and pharmacodynamic
EVs: Extracellular vesicles
OEVs: Organoid extracellular vesicles
SERS: Surface-enhanced Raman scattering
